# Phytobezoar: an unusual cause of intestinal obstruction

**DOI:** 10.2349/biij.1.1.e4

**Published:** 2005-07-01

**Authors:** HC Teng, O Nawawi, KL Ng, YI Yik

**Affiliations:** 1 Department of Biomedical Imaging (Radiology), Faculty of Medicine, University of Malaya, Kuala Lumpur, Malaysia; 2 Department of Surgery, University of Malaya Medical Centre, Kuala Lumpur, Malaysia

**Keywords:** Phytobezoars, small bowel obstruction

## Abstract

Small bowel phytobezoars are rare and almost always obstructive. There have been previously reported cases of phytobezoars in the literature, however there are few reports on radiological findings for small bowel bezoars. Barium studies characteristically show an intraluminal filling defect of variable size that is not fixed to the bowel wall with barium filling the interstices giving a mottled appearance. On CT scan, the presence of a round or ovoid intraluminal mass with a ‘mottled gas’ pattern is believed to be pathognomonic. Since features on CT scans are characteristics and physical findings are of little assistance in the diagnosis of bezoar, the diagnostic value of CT needs to be emphasised.

## INTRODUCTION

Phytobezoars are concretion of poorly digested fruit and vegetable fibres that are found in the alimentary tract, particularly orange pith or pulp in patients with history of surgery and persimmon in patients without previous surgery [1]. Persimmon contains a high concentration of tannin, a monomer that polymerise in the presence of gastric acid and the polymerized tannin then acts as a nucleus for bezoar formation.

Previous gastric resection or ulcer surgery such as partial gastrectomy or truncal vagotomy with pyloroplasty predisposes to bezoar. Other predisposing factors are ingestion of high fibre foods, abnormal mastication, diminished gastric secretion and motility, autonomic neuropathy in diabetic patients and myotonic dystrophy [2]. Bezoars are currently regarded as a sequel of gastric surgery and are included in the postgastrectomy syndromes. Incidence of post gastrectomy bezoar range between 5-12% [1]. In a normal stomach, vegetable fibres which cannot pass through the pylorus undergo hydrolysis within the stomach, which softens them enough to go through the small bowel. After gastric surgery, the gastric motility is disturbed and the gastric acidity is decreased, and the stomach may empty rapidly with an increased possibility of bezoar formation.

Normally found in the stomach, they may pass into the small bowel. Primary small bowel bezoar is very rare and is normally formed in patients with underlying small bowel disease such as diverticulum, stricture or tumour. Phytobezoar can also develop secondarily if there are areas of sufficient stagnation within a dilated bowel segment as may occur in patients with strictures caused by Crohn's disease, TB or previous surgery, or in patients with small bowel diverticula. In such cases, the bile constituents or calcium salts contribute to bezoar development [3].

We present an unusual case of small intestinal obstruction caused by phytobezoar and discuss the radiological investigations with special emphasis on CT appearance that leads to the diagnosis of this condition.

## CASE REPORT

An 81-year-old Chinese woman was referred to our centre from a private medical centre for further management of intestinal obstruction and pneumonia. She presented with one week history of epigastric discomfort associated with vomiting and abdominal distension. Her bowel habit was mildly altered but there was no history of passing blood per rectally. She denied any loss of weight or appetite. Interestingly she gave a history of eating a large amount of Chinese mushroom a few days prior to her presentation. Medically she was being treated for hypertension and congestive cardiac failure. Her past surgical history consists of a laparotomy performed 26 years ago and a right nephrectomy for renal stone performed 20 years ago. She was however unable to ascertain the reason for the laparotomy.

On admission, her vital signs were stable with BP of 200/90 and HR of 100/m. Chest auscultation revealed reduced air entry and crepitation at the right lower lobe. Her abdomen was soft but slightly distended. A mobile vague mass was palpable at the left iliac fossa. Bowel sound was sluggish and per-rectal examination revealed an empty rectum with no palpable mass. Her hernia orifice were normal.

Chest radiograph was done and showed right lower lobe consolidation with evidence of chronic obstructive airway disease and cor pulmonale. Echocardiogram showed evidence of cor pulmonale with no acute ischaemic changes. Her blood investigation results were unremarkable. Blood culture was positive for coagulase negative Staphylococcus. Urine culture and sputum AFB were negative. Her amylase level was normal.

**Figure 1:**
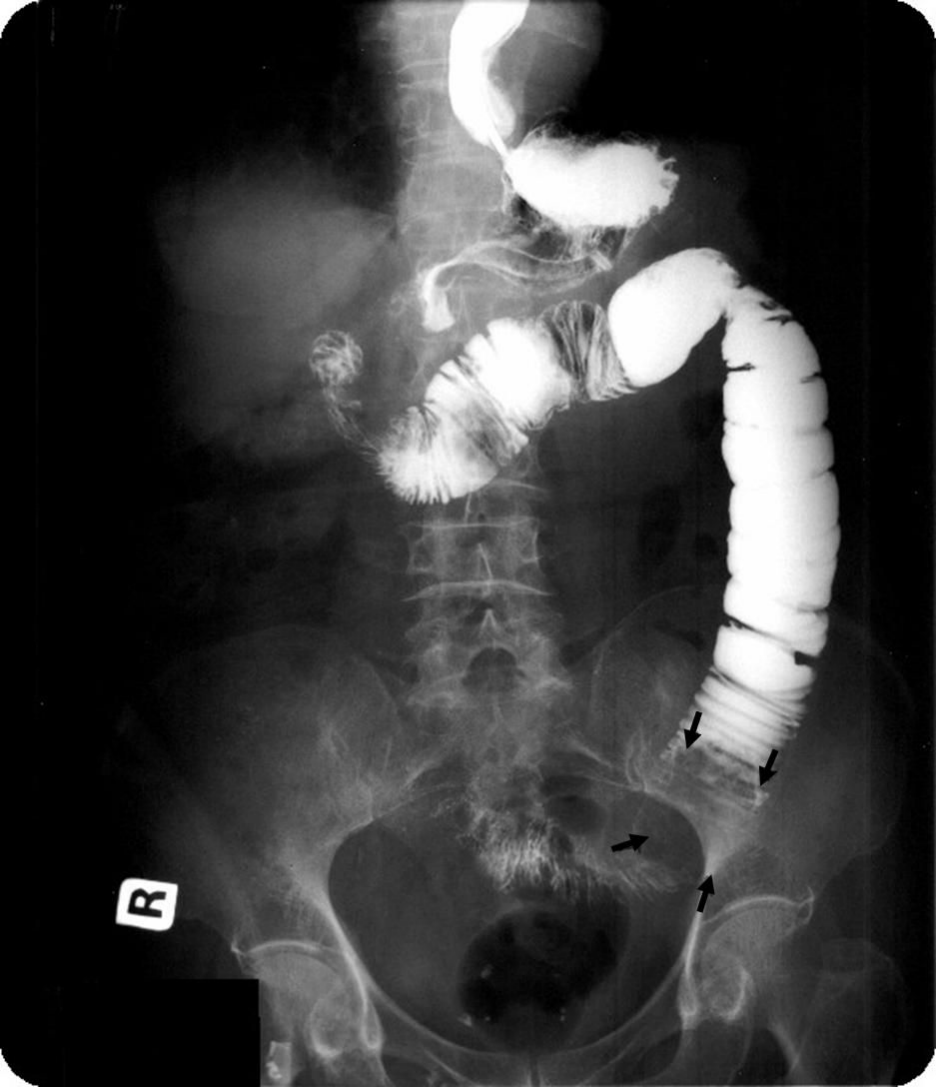
Barium study showing the filling defect in the jejunum with claw-like appearance suspicious of intussusception. The bowel loop distal to the filling defect is collapsed (arrow).

**Figure 2:**
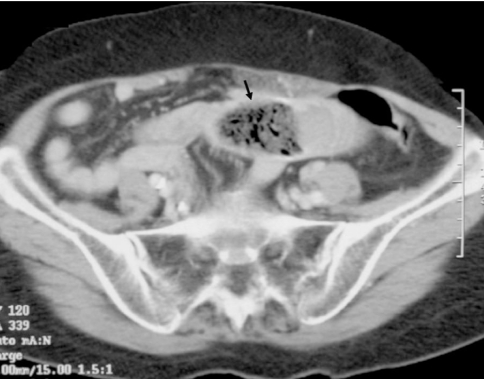
CT scan of the abdomen showing the intraluminal hypodense filling defect with mottled appearance (arrow).

Prior to admission to our hospital, the patient had a barium meal and follow-through examination and CT scan of the abdomen done at the referring hospital. The barium studies showed proximal small bowel dilatation with an oval intraluminal filling defect seen at the end of the dilated segment which was about 1 foot from the duodeno-jejunal junction. Also present were ‘coil spring’ and ‘claw’ appearance which led to the impression of an intussusception (Figure 1). It was reported that there was temporary hold up of contrast proximal to this level. Her stomach was of normal configuration and there was adequate gastric emptying. On CT scan of the abdomen, the filling defect seen in the barium study represented a hypodense intraluminal ovoid mass with mottled gas pattern noted within it (Figure 2). There was no obvious site of attachment to the bowel wall and there was no evidence of whorled appearance which we would expect to see in intussusception. No other intraluminal mass or polyps was found, excluding other causes of obstruction. Bowel distal to the mass was collapsed. The characteristic CT findings immediately alerted us to the diagnosis of a bezoar.

**Figure 3:**
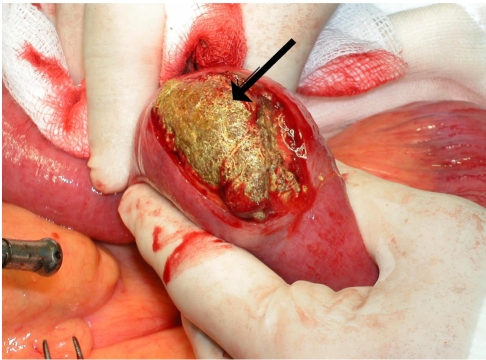
Intraoperative findings of phytobezoar in the jejunum (arrow).

Based on the radiological findings, clinical diagnosis of bezoar causing partial intestinal obstruction was made and an exploratory laparotomy with jejunotomy was performed on the second day of admission. The operation confirmed the clinical suspicion of a bezoar measuring 5 × 3 cm which was found at a distance of 34 cm from the duodeno-jejunal flexure (Figure 3). The jejunum was dilated and hypertrophied but there was no jejunal mass or polyps found. Incidental finding of left clear looking para-ovarian cyst measuring 4 × 4 cm led to left salpingo-oophorectomy at the same time.

Pathological report of the operative specimen was degenerate vegetable matter with no malignancy and a left paraovarian simple cyst. Post operative period was uneventful, where she was started on nourishing fluid and soft diet. She was discharged 10 days later with Tab Lisinopril 15mg bd, Tab Unasyn 375mg bd and Tab Bisolvent 8mg od. She was found to be well during follow up in surgical clinic one week later.

## DISCUSSION

Small bowel obstruction accounts for 20% of hospital admission. Common causes are adhesions, strangulated hernia, malignancy, volvulus and inflammatory bowel disease. Phytobezoars are rare, accounting for only 0.4 to 4% of all intestinal obstruction. No particular age or sex prevalence have been observed [4].

There are 4 types of bezoars. Phytobezoars are the most common, and are composed of vegetable matter such as celery, pumpkin, grape skin, prune and persimmons and it contains large amount of non-digestible fibres such as cellulose, hemicellulose, lignin and fruit tannins. Trichobezoars are gastric concretion of hair fibres which usually presents in patients with history of psychiatric predisposition and in children with mental retardation. Pharmacobezoars consist of medication bezoars, which in bulk will adhere, such as cholestyramine, kayexalate resin, cavafate and antacids. Lactobezoars are milk curd secondary to infant formula, described in low birth weight neonate fed on highly concentrated formula within the first week of life [5].

Primary small bowel bezoars almost always present as intestinal obstruction. They usually become impacted in the narrowest portion of the small bowel, the commonest site being the terminal ileum followed by the jejunum [6], as was found in our patient. It is interesting to note that more than half of cases of phytobezoars had history of previous gastric surgery [7]. Our patient gave a history of laparotomy done 26 years ago which could be gastrointestinal related surgery.

Plain radiograph typically shows a classic obstructive pattern. Occasionally we may be able to see the outline of bezoar, which is difficult to differentiate from abscess or faeces within the colon. Ultrasound has been used to detect bezoar. In a retrospective study done by Ripolles et al. [8], ultrasound was able to detect phytobezoar in 88% of patients with small bowel obstruction. Bezoar appears as a hyperechoic arc-like surface with acoustic shadowing on ultrasound, however this feature may make it difficult to differentiate it from gallstone which also has similar ultrasound characteristics.

Barium studies characteristically show an intraluminal filling defect of variable size that is not fixed to the bowel wall. Barium filling the interstices gives a mottled appearance similar to that of a villous tumour [2]. In our patient, the barium study showed an intraluminal filling defect with a claw appearance giving the impression of an intussusception. To the best of our knowledge this barium study finding has not been described by previous reports.

CT scan is fast becoming the first line examination for the evaluation of small bowel obstruction because it can exclude other causes of acute abdomen, differentiate between simple obstruction and strangulation, detect signs of concomitant intestinal ischemia and can accurately define the cause, degree and level of obstruction. The presence of round, non-homogenous mottled mass on CT enabled us to accurately diagnose bezoar as the cause of intestinal obstruction in our patient. Kim et al. found that in 11% of the cases, phytobezoar can appear as a soft tissue mass without gas making diagnosis difficult as it can resemble an intraluminal tumour or intussusception. They also described the presence of target sign found in 76% of their patients caused by mural edema or haemorrhage within the intestinal wall. The presence of this sign on CT indicates that the phytobezoar obstructing the bowel may have difficulty passing through the small bowel lumen. An encapsulating wall caused by a gel-like membrane covering the bezoar may also be seen on CT [3].

According to Andrus et al., endoscopy can definitively diagnose phytobezoar where it appears as a dark brown, green, black mass of amorphous material in gastric fundus, antrum or remnant stomach. They found that barium swallow identified only 25% of bezoars found endoscopically [5]. Endoscopy was not necessary in our patient as the bezoar was seen in the barium and CT examinations.

Small bowel bezoars are treated surgically. It is mandatory to explore the whole gastrointestinal tract in order to avoid synchronous bezoar and recurrence of intestinal obstruction due to retained bezoar. Other treatment options include enzymatic breakdown and endoscopic fragmentation for gastric bezoar [1,5].

Recurrence is common unless the underlying predisposing condition is corrected. Prevention includes avoidance of high fibre foods, introduction of prophylactic medication to improve gastric emptying and psychological or psychiatric follow up in patients with psychiatric disease [5]. In difficult recurrent cases, periodic endoscopy with repeated mechanical disruption is warranted.

In conclusion we present an uncommon case of small bowel obstruction caused by phytobezoar. CT findings can be diagnostic when there is presence of an intraluminal mass with mottled gas pattern at the site of obstruction associated with distal luminal collapse.
